# Effect of single intravenous injection of esketamine on quality of recovery during early period after modified radical mastectomy for breast cancer: A retrospective study

**DOI:** 10.12669/pjms.39.6.8057

**Published:** 2023

**Authors:** Guofang Fei, Wei Yan, Huaqi Yao

**Affiliations:** 1 Guofang Fei, Department of Anesthesiology, Huzhou Maternity & Child, Health Care Hospital, Huzhou 313000, Zhejiang Province, P.R. China; 2 Wei Yan, Department of Anesthesiology, Huzhou Maternity & Child, Health Care Hospital, Huzhou 313000, Zhejiang Province, P.R. China; 3 Huaqi Yao, Department of Anesthesiology, Huzhou Maternity & Child, Health Care Hospital, Huzhou 313000, Zhejiang Province, P.R. China

**Keywords:** Esketamine, Modified Radical Mastectomy for Breast Cancer, Quality of Recovery Score

## Abstract

**Objective:**

To assess the impact of a single esketamine intravenous (IV) injection on the quality of recovery during early period after modified radical mastectomy for breast cancer.

**Methods:**

This retrospective study included 80 patients who underwent modified radical mastectomy under general anesthesia in Huzhou Maternity & Child Health Care Hospital from March to October 2022. All patients were between 35 to 55 years, weighting between 45 and 70 kg and Grade-I or II according to the American Society of Anesthesiologists (ASA). Patients were grouped based on the type of pain management used. Patients (n=40) who were given 0.25 mg/kg esketamine single IV injection prior to completion of the surgery were assigned to Group-E, and patients (n=40) who were not treated with esketamine, comprised the control Group-C. Patients’ data, such as education years, operation time, blood loss, the 9-item Quality of Recovery (QoR-9) scores before the anesthesia induction (T_0_), one (T_1_) and two hours after the extubation (T_2_), the 40-item Quality of Recovery (QoR-40) scores one (D_1_) and two days after the surgery (D_2_), and the rate of adverse reactions were assessed in both groups.

**Results:**

Compared with T_0_, the QoR-9 scores at T_1_ and T_2_ were markedly lower in all patients. Compared to Group-C, the QoR-9 scores at T_1_ and T_2_ in Group-E were considerably increased (*P*<0.05). Similarly, the QoR-40 scores on D_1_ and D_2_ in Group-E were significantly higher compared to Group-C (*P*<O.05).

**Conclusion:**

Single intravenous injection of esketamine can improve the quality of the recovery of breast cancer patients during the early period after the modified radical mastectomy.

## INTRODUCTION

Modified radical mastectomy via axillary lymph node dissection preserves patient’s pectoralis major and minor muscles and medial and lateral pectoral nerves. This type of surgery is currently the most commonly used surgical procedure for breast cancer.^1^,^2^ However, postoperative inflammation, pain and limited shoulder movement can seriously affect patients’ postoperative recovery.^3^ The 9-item Quality of Recovery Scores (QoR-9) and 40-item Quality of Recovery Scores (QoR-40) are used to scientifically evaluate recovery quality of patients after the surgery.^4^,^5^

Ketamine has a positive effect in pain management and mood improvement.^6^,^7^ Ketamine injections that are currently used clinically in China are a equivocal racemic mixture of the two enantiomers (or optical isomers), S(+) ketamine and R(-) ketamine. Esketamine (S(+) ketamine) is twice as potent as racemic ketamine and has fewer side effect.^8^ This study evaluated the impact of a single IV injection of esketamine on the quality of early recovery in patients after modified radical mastectomy using the QoR-9 and QoR-40 scales.

## METHODS

This retrospective study was performed in Huzhou Maternity & Child Health Care Hospital. The target population was patients who underwent elective modified radical mastectomy for breast cancer in our hospital and consented to participate in the study. Patients were retrospectively allocated into two groups, Group-E and Group-C, n=40 each, based on the intraoperative pain management. A total of 80 ASA grade I-II patients, aged 35-55 years, weighting 45-70 kg, were included. Patients in Group-E were given 0.25 mg/kg esketamine single IV injection prior to completion of the surgery, while patients in Group-C did not receive the drug.

### Inclusion criteria:

- ASA grade I-II
- 35-55years, 45-70 kg
- Underwent elective modified radical mastectomy for breast cancer
- Complete clinical data

### Exclusion criteria:

- History of psychosis.
- Hypertension.
- Hyperthyroidism.
- History of allergy or addiction to narcotic drugs.
- Severe insufficiency of vital organs such as liver and kidney.
- Incomplete medical records

### Ethical Approval:

This study got the approval of the ethics committee of the Huzhou Maternity & Child Health Care Hospital (No. 2023-J-022, Date: 2023-03-02). Signed informed notification form was obtained from the participating patients.

Patients were admitted to the operating room and peripheral veins access was established. Continuous monitoring of blood pressure (BP), pulse oximetry (SpO_2_), electrocardiogram (ECG) and electroencephalographic bispectral index (BIS) was done. General anesthesia was induced by the sequential administering of 0.03 mg/kg midazolam, 0.2 mg/kg etomidate, 0.3 μg sufentanil/kg, 0.15 mg/kg cis-atracurium. Mechanical ventilation was maintained after the intubation. Intraoperatively, Propofol 4~6 mg·kg^-1^·h^-1^ and Remifentanil 0.1~0.3 μg·kg^-1^·h^-1^ were infused intravenously, and additional skeletal muscular relaxants were intermittently administered to maintain anesthesia with P_ET_CO_2_ 35~45mmHg, BIS 40~60.

Patients in the Group-E were administered a single IV dose of 0.25 mg/kg of esketamine, diluted in 0.9% saline before the extubation, 10 minutes before the end of the surgery. In cases of HR < 50 beats/minutes during the surgery, an IV injection of atropine 0.5mg was given to all patients; in cases of systolic blood pressure SBP drop > 20mmHg, phenylephrine 50μg was administered intravenously and repeated if necessary. After the operation, the analgesic pump was connected with the analgesic pump solution of 0.15 mg/kg Fentanyl + 8 mg Ondansetron + 1 μg/kg dexmedetomidine diluted to 100 ml with saline. The baseline dose of the analgesic pump was 2.0 ml/h and the single dose (PCA dose) was 2.0 ml/time with an interval of 15 minutes.

Patient’s data, such as years of education, duration of surgery, and intraoperative blood loss was recorded for both groups. Record of QoR-9 scores of all patients was done before the initiation of anesthesia (T_0_), one hour after the extubation (T_1_) and two hours after the extubation (T_2_). The QoR-9 score is used to measure the quality of recovery after general anesthesia, and contains nine items, ranging from the score zero to two, with a total score of 18. Higher scores indicate better quality of patients’ recovery from the general anesthesia.^9^ QoR-40 scores were recorded on post-operative day one (D_1_) and day two (D_2_) for both groups to assess quality of patients’ recovery after the surgery. The QoR-40 score includes 40 questions in five areas, such as physical comfort, emotional state, self-care, psychological support, and pain. Each question has a score of one to five. The total score ranges from 40 to 200. Greater total score indicates better recovery quality.^10^ The incidence of adverse reactions within 24 hours after the surgery was recorded for both groups.

### Statistical Analysis:

Analysis was done by SPSS 20.0 statistical software. Intergroup comparison of the normal distribution data was done by t-test and presented as mean ± standard deviation ( *x*±*s*). The statistical data were tested by χ2 test or Fisher’s exact probability method. P < 0.05 was statistically significant.

## RESULTS

As summarized in Table-I, general characteristics were similar in both groups of patients. Both groups had significantly lower QoR-9 scores at T1 and T2 than at T0 (P < 0.05). Group-E patients had significantly higher QoR-9 scores at T1 and T2 compared to Group-C (P < 0.05) (Table-II). All QoR-40 scores and total scores were significantly higher in Group-E patients at D_1_ and D_2_ compared to Group-C (*P*<0.05) (Table-III). The rate of adverse reactions within 24 h after the surgery was the same in both groups (P > 0.05) (Table-IV).

**Table-I:** Comparison of the general characteristics of patients in the two groups (*χ̅*±*S*).

Group-	Case (n)	Age (years)	BMI (kg/m^2^)	ASA grade I/II	Education years (years)	Operation time (min)	Bleeding volume (ml)
Group-E	40	45.6±4.7	22.5±2.8	36/4	12.7±2.5	112.6±17.5	99.1±25.3
Group-C	40	47.5±5.4	22.7±3.1	37/3	12.5±2.2	116.2±16.9	95.6±23.8
*t/*χ^2^		1.679	0.302	0.157	0.380	0.936	0.637
P value		0.082	0.745	0.692	0.753	0.325	0.655

**Table-II:** Comparison of QoR-9 scores between the two groups of patients (*χ̅*±*S*).

Group-	Case (n)	T_0_	T_1_	T_2_
Group-E	40	17.2±0.5	14.2±0.8^ab^	15.6±1.1^ab^
Group-C	40	17.1±0.3	13.7±1.0^a^	14.8±1.6^a^

***Note:*** Compared with T_0_,

a P<0.05; compared with Group-C, ^b^P<0.05.

**Table-III:** Comparison of post-operative QoR-40 scores between the two groups of patients (*χ̅*±*S*).

Time	Group-	Physical comfort	Emotional state	Self-care	Psychological support	Pain	Total score
D_1_	Group-E	53.3±2.8	42.1±3.1	13.4±2.6	35.5±1.0	33.8±2.3	178.5±7.6
	Group-C	51.2±2.2	36.6±2.3	11.5±3.5	32.4±1.5	31.6±3.4	163.4±8.2
	t value	3.730	9.012	2.756	10.876	3.390	8.541
	P value	<0.001	<0.001	<0.01	<0.001	<0.002	<0.001
D_1_	Group-E	55.4±2.1	43.3±2.6	18.4±3.1	36.7±0.8	34.2±2.1	188.7±5.7
	Group-C	52.7±1.8	40.2±2.5	15.9±3.2	33.1±1.3	32.7±2.6	174.5±6.3
	t value	6.174	5.436	3.549	14.916	2.839	10.571
	P value	<0.001	<0.001	<0.001	<0.001	<0.01	<0.001

**Table-IV:** Comparison of the incidence of adverse reactions in the two groups of patients (n%).

Group-	Case (n)	Hypertension	Tachycardia	Nausea and Vomiting	Nightmares and hallucinations
Group-E	40	1 (2.5%)	1 (2.5%)	3 (7.5%)	1 (2.5%)
Group-C	40	1 (2.5%)	1 (2.5%)	2 (5.0%)	0 (0)
*t/*χ^2^		0.000	0.000	0.213	1.013
P value		1.000	1.000	0.644	1.000

## DISCUSSION

The primary finding of this study is that single intravenous injection of esketamine can improve the quality of recovery of breast cancer patients during the early postoperative period after the modified radical mastectomy.

In the current study, the QoR-9 and QoR-40 scores are designed as the primary outcome measure. QoR-9 scores system is a nine-item patient-rated score of self-perceived comfort that assesses the patient’s quality of recovery after the general anesthesia using the following criteria: anxiety and fear; level of need for support from others; level of understanding of instructions; level of simple living; level of gastrointestinal discomfort and self-toileting; level of ease of breathing; level of headache, back pain and muscle pain; level of nausea and vomiting; and level of severe pain or persistent moderate pain.^9^ The QoR-40 score has strong clinical applicability and can effectively reflect the postoperative recovery situation. The total score of QoR-40 is 40-200 points, representing very poor to excellent postoperative recovery.

Modified radical mastectomy for breast cancer can have both physical and psychological impact on the patient, as it is associated with considerable postoperative pain and emotional distress due to cosmetic defects. Studies show that patient’s distress may significantly affect wound healing.^11^,^12^ Therefore, timely interventions to promote recovery are particularly important. Ketamine has anti-inflammatory and neuroprotective proprieties,^13^,^14^ with significant analgesic effects at low doses and narcotic effects at high doses.^15^ Esketamine is twice as potent as racemic ketamine. Similarly, to the study by Imbellon et al,^16^ we used a single IV injection of 0.25 mg/kg of esketamine as a subanesthetic dose.

Patients that were administered esketamine (Group-E) had better self-perceived comfort, need for support from others, simple living, and pain-related scores at T_1_ and T_2_ as opposed to the control group. The QoR-9 scores of patients in Group-E were significantly elevated, suggesting that a single subanesthetic dose of esketamine could significantly improve patients’ comfort levels during the early postoperative stage, probably due it its anti-inflammatory and analgesic effect. A single subanesthetic dose of esketamine was associated with increased patient self-perceived comfort after the surgery.

Our results are consistent with the observations of Cao et al.^17^ Esketamine can prevent opioid-induced nociceptive hypersensitivity, reduce the required amount of postoperative analgesia, and prolong its duration.^18^ Perioperative intravenous subanesthetic doses of ketamine also lower the occurrence rate of chronic postoperative pain.^19^ Post-mastectomy pain syndrome (PMPS) is present in approximately 20% to 68% of patients after modified radical mastectomy for breast cancer and is generally thought to be caused by postoperative hyperexcitability of the associated spinal dorsal horn neurons, acting mainly through N-methyl-D-aspartate (NMDA) receptor mediation.^20^ Esketamine can control chronic pain by effectively affecting NMDA receptor expression and structural and functional hyperactivation of microglia, astrocytes, and synapses.^21^

We have observed that the pain scores and physical comfort scores in the QoR-40 at D_1_ and D_2_ were markedly greater in Group-E patients compared to the patients in the control group.

Previous studies also demonstrated the marked antidepressant effect of subanesthetic doses of ketamine.^22^ Esketamine was shown to improve depressive symptoms most significantly at two hours and four hours after administration, and its antidepressant effect persisted for about seven days after discontinuation of the drug.^23^,^24^ Esketamine administration led to a rapid improvement in the mental and motor functions of the patients after the surgery.^25^ Our results showed that the emotional state, psychological support scores and self-care scores at D_1_ and D_2_ were significantly greater in Group-E compared to Group-C patients, as indicated by better QoR-40 scores. The final QoR-40 total score was also markedly higher in Group-E patients.

The common adverse effects of esketamine include increased BP and HR, psychiatric symptoms such as anxiety, nightmares, hallucinations, and dissociation, as well as nausea and vomiting. However, these adverse effects are transient and dose-dependent.^26^ No differences in the rate of postoperative adverse effects was detected between the groups in this study when esketamine was administered at 0.25 mg/kg, indicating that a single postoperative sub-anesthetic dosage of esketamine did not increase the incidence of adverse effects.

### Limitations:

This is a retrospective study with a small sample size. Further prospective studies with larger sample sizes are needed to confirm our results. Only 0.25 mg/kg of esketamine was administered to the patients. Further studies are needed to determine whether this is the optimum dose. Second, esketamine was given before the extubation, 10 min before the end of the surgery. Further studies should determine the optimal timing for the administration of esketamine.

## CONCLUSION

A single postoperative intravenous injection of esketamine is simple and easy to administer and can lead to significantly improved quality of early recovery after breast cancer surgery. Single postoperative IV injection of esketamine, thus, may be safely used in clinical practice.

### Authors’ contributions:

**GF:** Conceived and designed the study.

**WY** and **HY:** Collected the data and performed the analysis.

**GF:** Was involved in the writing of the manuscript and is responsible for the integrity of the study.

All authors have read and approved the final manuscript.
